# Causal Inference and Survey Data in Paediatric Epidemiology: Generalising Treatment Effects From Observational Data

**DOI:** 10.1111/ppe.70042

**Published:** 2025-07-14

**Authors:** Lizbeth Burgos‐Ochoa, Felix J. Clouth

**Affiliations:** ^1^ Department of Methodology and Statistics Tilburg University Tilburg the Netherlands; ^2^ Department of Population, Policy & Practice Great Ormond Street Institute of Child Health, University College London London UK

**Keywords:** G‐computation, inverse propensity weighting, second‐hand smoke, survey weights, targeted maximum likelihood estimation, transportability

## Abstract

**Background:**

Survey data are essential in paediatric epidemiology, providing valuable insights into child health outcomes. The potential outcomes framework has advanced causal inference using observational data. However, traditional design‐based adjustments, especially sample weights, are often overlooked. This omission limits the ability to generalise findings to the broader population.

**Objective:**

This study demonstrates three approaches for estimating the population average treatment effect (PATE) in a practical example, examining the impact of household second‐hand smoke (SHS) exposure on blood pressure in school‐aged children.

**Methods:**

Using data from the National Health and Nutrition Examination Survey (NHANES) 2017–2020, we assessed the effect of household SHS exposure, a non‐randomised treatment, on blood pressure in school‐aged children. We applied estimators based on Inverse Probability of Treatment Weighting (IPTW), G‐computation, Targeted Maximum Likelihood Estimation (TMLE), and regression adjustment. Models without adjustments were run for comparison. We examined point estimates and the efficiency of the estimates obtained from these methods.

**Results:**

The largest differences were observed between the unadjusted regression models and the fully adjusted methods (IPTW, G‐computation, and TMLE), which account for both confounding and survey weights. While the inclusion of the sample weights leads to wider confidence intervals for all methods, G‐computation and TMLE showed comparatively narrower confidence intervals. Confidence intervals for the models not adjusted for sample weights were likely underestimated.

**Conclusions:**

This study highlights the important role of sample weights in causal inference. Generalisability of the average treatment effect as estimated on data sampled using common survey designs to a defined population requires the use of sample weights. The estimators described provide a framework for incorporating sample weights, and their use in health research is recommended.

## 1 Background

Population health surveys have long been essential for collecting and accessing health data for research and public health monitoring. In recent years, they have expanded beyond self‐reported health measures to include environmental and social exposures, physical measures and biomarkers [1]. These surveys use complex multi‐stage designs with planned analytical adjustments to ensure that results can be generalised to the broader population [2], a crucial feature for research aimed at guiding public health interventions. The use of health surveys in perinatal and paediatric research has grown substantially.

Epidemiological research often seeks to answer causal questions, such as investigating the effects of indirect tobacco smoke exposure on children's health outcomes. When randomised controlled trials are not feasible, researchers turn to observational data, such as survey data, to address these questions [3]. While survey data are frequently used to estimate exposure–outcome relationships with causal intent, evidence derived from these studies is often framed as associational, even when researchers take steps aligned with causal inference principles. This cautious interpretation may stem from concerns about unmeasured confounding and the inherent limitations of observational data. The potential outcomes (PO) framework has allowed researchers to define the causal effect they aim to identify (the estimand) and to determine the assumptions necessary to estimate this effect without bias [4]. A commonly used estimand is the Average Treatment Effect (ATE), which pertains either to the sample in which it was estimated or to a broader population for which the observed sample is a simple random sample [5]. The PO framework has facilitated the development of robust methods, each employing different strategies to address these assumptions and reduce bias.

Causal inference literature has primarily focused on improving internal validity, ensuring that the resulting estimate reflects an unbiased causal effect that cannot be attributed to other factors, e.g., as confounding [5, 6]. However, comparatively less attention has been given to external validity, referred to as “transportability” in causal inference literature, which addresses the ability to generalise findings beyond the study sample [5]. This topic has primarily been explored in the context of randomised trials, for example, the work by Westreich et al. [7], though it is equally relevant for observational studies. While not all studies aim for generalisability, many do [8], and results can be biased if the sample is not representative of the target population [6]. In nationally representative health surveys, the sample is often obtained using complex multi‐stage designs where selection probabilities vary due to factors such as oversampling of minority groups, clustering, stratification and non‐response [2]. Consequently, the sample is not a simple random sample of the population, and the ATE in the sample does not necessarily reflect the population average treatment effect (PATE) [6]. When the PATE is the estimand of interest, methods that address both internal and external validity bias are essential.

Traditional survey methodology offers corrections, in the form of sample weights, to enhance external validity [9]. This procedure, known as inverse probability weighting (IPW) in the survey literature, is essential for valid population estimates [9]. However, these adjustments are often overlooked when applying popular causal inference methods in epidemiology [6, 10]. Recent simulation studies focused on the use of widespread causal inference approaches have demonstrated the bias that can arise from omitting sample weights in the analysis when the aim is to estimate the PATE [6, 10].

Among causal inference approaches based on the POs framework, inverse probability of treatment weighting (IPTW) is the most commonly applied approach for incorporating survey weights, as demonstrated in several applied studies [11, 12, 13]. However, IPTW is known to be statistically inefficient, often resulting in large standard errors due to high‐variance weights [14]. Alternative approaches, such as estimators based on the G‐computation (G‐formula) and doubly robust methods like targeted maximum likelihood estimation (TMLE), may offer solutions to this issue [6]. The underuse of sample weights in combination with other methods to account for confounding may stem from a lack of clear guidance in the literature [6].

Although the survey and causal inference literature have largely developed independently, they have notable connections. For instance, IPW, originally developed to address unequal selection probabilities into the survey sample, laid the foundation for IPTW, one of Robins' influential G‐methods [15].

This paper demonstrates three PATE estimators based on IPTW, G‐computation and TMLE. Using NHANES (2017–2020) data, we estimate the effect of household second‐hand smoke (SHS) exposure on blood pressure in school‐aged children. Our example illustrates the methods and offers practical guidance for applying causal inference with survey data to generalise findings.

## 2 Methods

### 2.1 Potential Outcomes Framework

The causal question ‘What is the effect of being exposed to SHS on school‐aged children's BP?’ can be framed within the Neyman‐Rubin POs framework [4, 16]. We denote the outcome BP as Y and the exposure of interest as SHS, which can take the (observed) values *SHS* = 1 (exposed to SHS) or *SHS* = 0 (not exposed to SHS). POs refer to the outcomes that would have been observed under hypothetical scenarios, i.e., alternative stories. If we would set the exposure shs hypothetically (potentially counter to the fact) to 1 for all cases, the PO is denoted as $Y_{\mathrm{shs}=1}$ and if shs, potentially counter to fact, is set to 0, as $Y_{\mathrm{shs}=0}$. Note that we use lower case to denote hypothetical exposure settings, while upper case denotes observed exposure values. The estimand can then be defined as the ATE, which corresponds to the difference in the mean outcome values between these two hypothetical scenarios $E\left[Y_{\mathrm{shs}=1}-Y_{\mathrm{shs}=0}\right]$.

**FIGURE 1:**
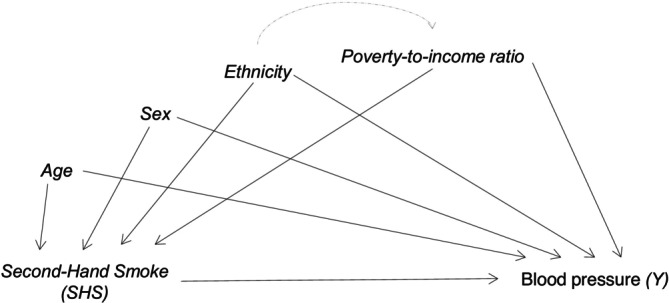
Directed acyclic diagram (DAG) for the hypothesised relationships between second‐hand smoke (SHS) and blood pressure confounded by child's age, sex, ethnicity and poverty‐to‐income ratio.

The directed acyclic graph (DAG) in Figure 1 illustrates the hypothesised relationships between exposure, outcome and potential confounding variables (age, sex, ethnicity and poverty‐to‐income‐ratio). For simplicity, in the text we refer to the set of observed confounders as L. For any estimate to be interpreted causally (ATE, PATE, etc.), certain internal validity assumptions (known as causal identification assumptions) are required: exchangeability, consistency and positivity. A summary of these assumptions can be found in Table 1.

**TABLE 1:** Internal validity (causal identification) assumptions.

Causal identification assumptions are essential for estimating any causal effect, not solely those derived from approaches based on the potential outcomes' framework; however, this framework made these assumptions explicit. A description of each assumption can be found below

Assumption	Description
(Conditional) Exchangeability	Assumes that groups can be interchanged without influencing the study outcome, provided confounding is controlled. Confounders are variables that are related to both exposure and outcome. For example, for the effect of SHS on BP, researchers adjust for measures related to economic resources/poverty. However, in this analysis, it is likely that some relevant confounders are missing. For instance, parental stress may influence both SHS exposure (e.g., parents under stress may be more likely to smoke at home) and child's blood pressure through other mechanisms such as disrupted sleep patterns or altered parenting behaviours. The absence of such confounders in the model may introduce residual confounding. Sensitivity analyses for unmeasured confounding are available, including methods for continuous, binary and time‐to‐event outcomes (see Comment section)
Consistency	This assumption connects observed outcomes with potential outcomes, requiring that an individual's observed outcome under an exposure is the same as if that exposure were hypothetically assigned. The consistency assumption requires a well‐defined exposure, meaning there should be no multiple ‘versions’ of it. In our study, household SHS exposure is defined based on the presence of at least one smoker in the household. However, variations in smoking frequency, intensity, and household ventilation (e.g., outdoor smoking areas) may introduce different versions of the exposure, affecting interpretation. Thus, our estimates should be understood as an average effect across these variations
Positivity	Assumes that each covariate combination has a nonzero probability of observing all exposure levels. This requires sufficient variation in exposure within all strata of confounders to allow for a meaningful estimation of effects. If certain subgroups (e.g., children from high‐income households) are never exposed to SHS, it becomes unfeasible to estimate the effect within those groups, leading to bias

As visualised in our DAG (Figure 1), the exposure–outcome relationship is confounded by a set of covariates, L. These confounders are common causes of both exposure and outcome. To estimate the ATE from observational data, it is necessary to adjust for this confounding. In this paper, we implement three commonly used approaches: IPTW [15], the G‐computation [17] and TMLE [18]. We then demonstrate how each method can be extended to incorporate survey sample weights for estimating the PATE.

### 2.2 IPTW: An Approach Based on Propensity Scores

IPTW is a method that accounts for confounding by statistically ‘breaking’ the relationship(s) between confounders and exposure [19]. It does so by reweighting individuals in the observed dataset based on how likely they were to receive the exposure, given their observed characteristics. This likelihood is captured by the propensity score, which represents the probability of receiving the exposure conditional on observed confounders [20]. To obtain these scores, one must model the exposure assignment mechanism, that is, estimate how the exposure (e.g., SHS) is assigned across individuals as a function of their covariates.

In the first step, the propensity score for each individual, denoted as $\pi_{i}=P\left(\mathrm{SHS}=1|L\right)$ is estimated using logistic regression, where *SHS* is the binary exposure variable, and ***L*** is the set of confounding variables. The propensity score summarises an individual's likelihood of being exposed based on their characteristics. The propensity score entails all the information of the confounders in a single score.

In the second step, IPTW weights are calculated as the inverse of the propensity score: exposed individuals receive a weight of $1/\pi_{i}$, and unexposed individuals receive a weight of $1/1-\pi_{i}$ [14, 21]. This means that individuals whose exposure status is rare given their covariates (e.g., exposed despite low predicted probability) are upweighted, while common cases are downweighted. This reweighting creates a pseudo‐population in which the distribution of measured confounders is balanced across exposure groups, emulating a randomisation [21].

In the third step, the ATE is estimated as the difference in average outcomes between the weighted exposure groups. Finally, 95% confidence intervals can be calculated using model‐robust standard errors, such as those implemented in the *survey* package in R. Compared to approaches like propensity score matching, IPTW uses the full sample, which can offer greater statistical power [22, 23].

### 2.3 The G‐Computation

The G‐computation accounts for confounding by statistically “breaking” the confounder‐outcome relationship [19]. To do so, the G‐computation requires modelling the outcome mechanism [20], that is, the relationship between the outcome and both the exposure and confounders, expressed as $E\left(Y|\mathrm{SHS},L\right)$. The G‐computation is the most commonly used approach to estimate the G‐formula [24], which is a mathematical expression for the expected POs across exposure conditions [20]. While the G‐formula is a theoretical quantity, G‐computation provides its implementation, typically using parametric (regression) models to estimate the outcome mechanism.

The G‐computation was originally proposed by Robins for longitudinal scenarios where confounders are affected by previous exposure [17]. In a cross‐sectional setting, however, the G‐computation is equivalent to standardisation [25], a widely used method in epidemiology, with the total sample serving as the standard population [17]. This approach estimates what the outcome would have been under different exposure scenarios for each individual in the dataset. That is, the G‐computation estimates the ATE $E\left[Y_{\mathrm{shs}=1}-Y_{\mathrm{shs}=0}\right]$ as $\int_{L}E\left(Y|\mathrm{shs}=1,L\right)f\left(L\right)dL-\int_{L}E\left(Y|\mathrm{shs}=0,L\right)f\left(L\right)dL$. The process involves the following steps:

In the first step, a (regression) model for the outcome conditional on exposure status and the confounders is fitted as $E\left(Y|\mathrm{SHS},L\right)$. In step 2, after fitting the outcome model, counterfactual outcomes are generated by setting everyone's exposure to shs=1 and then to shs=0, respectively, and predicting their outcomes under each scenario. This process is repeated over many Monte Carlo (MC) iterations to account for simulation variability. In the third step, resulting counterfactuals are averaged across individuals and MC iterations to estimate the ATE as the difference in mean outcomes between the two exposure scenarios. Last, 95% confidence intervals of the ATE are obtained via non‐parametric bootstrapping [24].

### 2.4 Targeted Maximum Likelihood Estimation

TMLE builds upon both IPTW and G‐computation by simultaneously ‘breaking’ the exposure–confounder and outcome–confounder relationships to address confounding [19]. It integrates information from both the propensity score (as in IPTW) and the outcome model (as in G‐computation).

In the first step, TMLE requires modelling the exposure mechanism $P\left(\mathrm{SHS}=1|L\right)$ and the outcome mechanism $E\left(Y|\mathrm{SHS},L\right)$. In the second step, counterfactual outcomes are predicted using the regression parameters from the outcome model $E\left(Y|\mathrm{SHS},L\right)$ as is done in the G‐computation. The third step is referred to as the ‘targeting step’ where a propensity score is obtained as it is done with IPTW. This propensity score is then used in step four to construct a ‘clever covariate’ $H_{i}={\mathrm{shs}}_{i}\frac{1}{\pi_{i}}-\left(1-{\mathrm{shs}}_{i}\right)\frac{1}{1-\pi_{i}}$ [18]. This clever covariate encodes the information from the exposure mechanism. In step 5, the clever covariate $H_{i}$ is used to update the initial outcome predictions, those from $E\left(Y|\mathrm{SHS},L\right)$, via a targeting step. In step 6, the updated estimate of $E\left(Y|\mathrm{SHS},L\right)$ is used to simulate updated sets of counterfactual outcomes and the differences between these counterfactuals are calculated. The ATE is then computed as the mean difference in outcomes under the shs=1 and shs=0 scenarios. In the last step, 95% confidence intervals can be obtained using targeted bootstrapping, a resampling method developed to be applied with TMLE [18].

TMLE has two key advantages: it optimises the bias‐variance tradeoff through the targeting step, and it is doubly robust. This means it yields consistent estimates if either the outcome model or the exposure model is correctly specified, but not necessarily both [18, 19].

### 2.5 From ATE to PATE

The ATE, as discussed earlier, represents the expected effect of an exposure in the study sample, or in the broader population if the sample is a simple random sample. In contrast, the PATE adjusts for differences between the sample and population, typically using survey weights. The ATE in our study reflects the effect of SHS exposure on blood pressure within the NHANES sample, while the PATE generalises this effect to the full population of U.S. children. A related estimand, the PATE among the exposed, focuses on those already experiencing SHS exposure and may be more relevant for targeted public health interventions. Policymakers might prioritise this when designing regulations, whereas the PATE offers population‐wide insights. Although all three causal inference methods described above can estimate the sample ATE, survey weights are essential for unbiased PATE estimation [6].

Sample weights, denoted as $\omega^{\Delta_{\mathrm{svy}}=1}$, adjust for the probability of selection and inclusion in the final dataset ($\Delta_{\mathrm{svy}}=1$), accounting for sampling design, non‐response and other post‐collection adjustments [26]. Table 2 summarises how these weights are incorporated into IPTW, G‐computation and TMLE; full details are provided in Table S1.

**TABLE 2:** Description of estimators for the population average treatment effect (PATE) using survey weights.

*Inverse Probability of Treatment Weighting (IPTW)* In IPTW, to account for both confounding and sampling bias, the survey weights $\omega^{\Delta_{\mathrm{svy}}=1}$ are multiplied by the IPTW treatment weights, creating a final set of analysis weights used to estimate the PATE. Since this is the most straightforward way to incorporate survey weights into analyses using methods from the potential outcomes framework, it is by far the most widely used approach in applied research. *G‐computation* To estimate the PATE using the G‐computation, we first fit a model for the outcome (blood pressure) while incorporating survey sample weights $\omega^{\Delta_{\mathrm{svy}}=1}$ to account for the survey sample design. Then, this model is used to predict counterfactual outcomes under different exposure conditions (SHS vs. no SHS) through Monte Carlo (MC) simulation. The treatment effect is obtained by calculating the difference between these counterfactual outcomes for each individual. Finally, to ensure the estimate reflects the target population, we take a weighted average of these differences using the survey sample weights. *Targeted maximum likelihood estimation (TMLE)* The TMLE estimate of the PATE follows a similar approach to the g‐formula, starting with the estimation of counterfactual outcomes under different exposure conditions (SHS vs. no SHS). However, TMLE includes an additional targeting step, which refines these initial estimates by incorporating information from both the treatment and outcome models. This adjustment is done using a specialised covariate, H, that incorporates the combined survey ($\omega^{\Delta_{\mathrm{svy}}=1}$) and IPTW weights, ensuring proper adjustment for both confounding and selection bias. Finally, the PATE is obtained by taking the weighted average of these refined treatment effects across the sample, using the survey sample weights to generalise the estimate to the target population.

### 2.6 Practical Example

To demonstrate IPTW, G‐computation and TMLE for estimating the PATE with survey data, we replicate the analytical setup from Liu et al. [27], who studied the effect of SHS exposure on children's blood pressure using NHANES 2007–2012. We use the same covariates and exposure definition but apply them to a newer NHANES wave (2017–2020), focusing on continuous systolic and diastolic BP as outcomes. For clinical interpretation of the findings, we refer readers to the original study.

#### 2.6.1 Data Source

The National Health and Nutrition Examination Survey (NHANES) [28] is a cross‐sectional survey assessing the health and nutrition of the non‐institutionalised US population. It collects demographic, health and biological data through interviews and physical exams in mobile examination centres (MECs) [28, 29]. NHANES uses a complex multi‐stage probabilistic sampling design and requires the use of sample weights to ensure representativeness [30]. We used data from the 2017–2020 cycle, including children aged 8–11 from the MEC subsample, as blood pressure was not measured in younger children.

#### 2.6.2 Outcomes

Systolic and diastolic blood pressure (SBP and DBP) were measured during MEC exams, and the average of available readings was used (mmHg) in the analysis [27].

#### 2.6.3 Exposure

Household SHS exposure was assessed using the NHANES smoking questionnaire, coded as 1 if any household member smoked and 0 otherwise. Further details are in Table S2.

#### 2.6.4 Statistical Analysis

To compare estimators, we included the following reference regression models: unadjusted, confounder‐adjusted without sample weights and weighted using sample weights without confounder adjustment. A fully adjusted model with both confounders and sample weights was also included. Models were adjusted for age, sex, ethnicity and poverty (see Table S2), excluding potential mediators like obesity to avoid overadjustment [31]. All weighted models incorporated NHANES design variables (see https://wwwn.cdc.gov/nchs/nhanes/tutorials/weighting.aspx for further guidance) using the *survey* package [32] in R.

The PATE was estimated using IPTW, G‐computation and TMLE. IPTW used logistic regression for propensity scores. G‐computation applied linear regression with 250 Monte Carlo iterations, and TMLE (via the *tmle* R package [33]) used the same model setup. For comparability, exposure and outcome models were identical across methods. Both G‐computation and TMLE generated 95% confidence intervals using the percentile method with 10,000 bootstrap iterations. The *tmle* package also supports machine learning‐based model estimation.

#### 2.6.5 Missing Data

All cases with missing outcome (*n* = 195) or exposure (*n* = 43) data were excluded prior to analysis (see Figure S1). The final dataset used for modelling contained no missing data in any of the remaining variables.

#### 2.6.6 A Note on Collapsibility

Adjusted regression models estimate conditional effects, reflecting the impact of the exposure while holding covariates constant. In contrast, IPTW, G‐computation, and TMLE estimate the ATE, a marginal effect, representing the expected outcome difference if the entire population were exposed vs. unexposed. Comparisons between conditional estimates and marginal estimates are valid only in collapsible settings, such as with linear models (as the ones used in this paper for continuous outcomes) and in the absence of effect modification. In non‐collapsible contexts (e.g., odds ratios or hazard ratios), these comparisons may be misleading. For further discussion, see Mood [34].

## 3 Results

In total, there were 1009 cases available for analysis. The sample flow diagram is presented in Figure S1, and Table 3 provides the descriptive statistics with and without sample weights.

**TABLE 3:** Descriptive Characteristics (NHANES 2017–2020 MEC cases, age 8–11 years).

Characteristic	Unweighted^a^		Weighted^a^	
	Mean (SE)^b^, ^c^	Percent^b^, ^c^	Mean (SE)^b^, ^c^	Percent^b^, ^c^
Average systolic blood pressure (BP)	100 (0.28)		100 (0.39)	
Average diastolic BP	60 (0.24)		59 (0.29)	
Age in years	9.55 (0.04)		9.57 (0.05)	
Second‐hand smoke exposure		31.4		24.7
Sex (Female)		51.1		50.6
Ethnicity				
Mexican American		17.3		17.0
Other Hispanic		9.7		9.4
Non‐Hispanic White		28.9		48.7
Non‐Hispanic Black		26.8		13.4
Other race—including multi‐racial		17.2		11.5
Poverty status				
Below poverty threshold		37.3		27.1
Near poverty		34.9		32.8
Above poverty threshold		20.8		35.6

^a^ Unweighted values represent raw sample estimates; weighted values incorporate NHANES sample weights to account for the complex survey design, ensuring representativeness of the U.S. noninstitutionalised population.

^b^ The total analytic sample includes 1009 children aged 8–11 years from the MEC subsample of NHANES 2017–2020. The sample size is the same for all variables in the table.

^c^ There were no missing data for variables included in this table. Cases with missing outcome or exposure data were excluded from the analysis as shown in Table S1.

**FIGURE 2:**
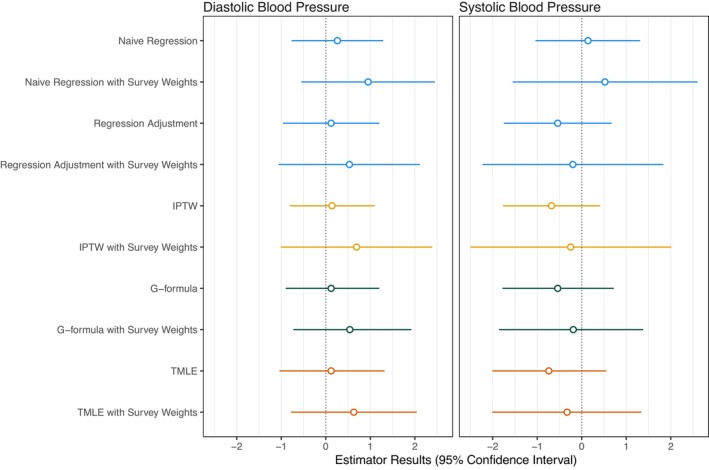
Estimated effects of household second‐hand smoke (SHS) exposure on blood pressure across different estimators. This figure displays the estimated effects (point estimates and 95% confidence intervals) of household SHS exposure on diastolic and systolic blood pressure using various estimators: Naive (unadjusted regression model); Regression model adjusted for survey weights only; Regression model adjusted for confounders only; Regression model adjusted for both confounders and survey weights; IPTW (Inverse Probability of Treatment Weighting) without survey weights); IPTW incorporating survey weights; G‐computation without survey weights; G‐computation with survey weights; TMLE (Targeted Maximum Likelihood Estimation) without survey weights; TMLE incorporating survey weights.

Figure 2 shows the results for all the estimators, including those that do not account for the sampling weights. Results are shown for a set of regression models (a naïve model without adjustment for confounding and without sample weights, a model without adjustment for confounding and including sample weights, a model with adjustment for confounding and without sample weights, and a model with adjustment for confounding and including sample weights) and the three causal inference approaches described above, i.e., IPTW, the G‐computation and TMLE, all with and without the inclusion of sample weights. The full numerical results are available in Table S3.

Differences were observed between the estimators, including and excluding the sample weights. Estimators, including the sample weights, yielded similar results as observed in Figure 2. The confidence intervals for the G‐computation were the narrowest, indicating greater efficiency (i.e., more precise estimates), while those for the IPTW approach were the widest. We observe that ignoring the sample weights resulted in confidence intervals that are comparatively narrow to the ones including the sample weights. Moreover, it is noticeable that the results from the IPTW estimator resulted in wider confidence intervals than the results from the G‐computation and TMLE. This aligns with existing literature, which describes the use of IPTW with survey weights as an inefficient estimator, often producing higher variance than other approaches.

## 4 Comment

### 4.1 Principal Findings

We demonstrated the use of three causal estimators of the PATE, i.e., IPTW, the G‐computation and TMLE, to calculate the effect of SHS on BP in school‐aged children. The estimators that accounted for both internal and external validity biases yielded similar results. For DBP, the point estimate shows a small effect of SHS. For systolic blood pressure (SBP), the point estimates are close to the null. However, for both outcomes, limited conclusions can be derived due to the large uncertainty of the estimates. In this analysis, G‐computation demonstrated greater precision, with the narrowest confidence intervals, while the IPTW method showed the widest confidence intervals.

### 4.2 Strengths of the Study

This study has several notable strengths. First, this study advances causal inference with observational data by demonstrating practical applications of causal estimators of the PATE. Second, it highlights the importance of clearly defining the target estimand—whether the ATE or the PATE—and the corresponding methodological implications. Estimating the PATE requires addressing both confounding and selection into the observed sample through appropriate weighting. Moreover, by using the publicly available NHANES dataset, the study supports reproducibility and encourages broader adoption of these methods.

### 4.3 Limitations of the Data

Our study is limited by a small sample size, as we used only the 2017–2020 NHANES cycle for demonstration, though more data are available. While our estimates align with previous studies, they remain imprecise. Including earlier NHANES cycles (as in Liu et al.) [27] could improve efficiency. Additionally, SHS exposure was based on self‐reported household smoking, which may lead to underreporting and misclassification, potentially underestimating true exposure levels.

Regarding causal identification assumptions, conditional exchangeability requires adjusting for all factors influencing both exposure and outcome. While we account for confounders commonly used in literature, residual confounding is likely. Although this assumption cannot be directly tested, sensitivity analyses can assess the potential impact of unmeasured confounders on the results. Lastly, we observed no indications of positivity violations (i.e., extreme weights), though the consistency assumption may be affected by various potential versions of SHS exposure as mentioned in Table 1.

### 4.4 Interpretation

This study reinforces the importance of clearly defining the estimand in epidemiological research. Aligning the study target with research objectives is essential for designing appropriate methods and ensuring conclusions remain consistent [35]. When estimating the PATE using survey data, methods must address both internal and external validity by adjusting for unequal selection probabilities. If the ATE is the focus, internal validity adjustments suffice, but conclusions should not imply generalisability without strong assumptions.

The wide 95% CI for IPTW reflects known efficiency issues, which worsen when multiplying IPTW and survey weights [14]. TMLE may inherit this inefficiency through the clever covariate in its targeting step [6]. Weight truncation is a potential solution but may introduce bias, and its impact when including sample weights remains underexplored. In contrast, G‐computation applies sample weights differently, avoiding weight multiplication, which may explain its superior efficiency in this study.

We used a non‐parametric bootstrap to construct confidence intervals for the g‐computation estimates. While commonly used, this method does not fully account for NHANES's complex design. Design‐aware alternatives like the Rao‐Wu‐Yue bootstrap or replicate‐weight methods preserve clustering and stratification, yielding more conservative standard errors [36].

## 5 Conclusions

Our study applied IPTW, G‐computation and TMLE to estimate the PATE of SHS exposure on children's blood pressure, emphasising the role of sample weights for generalisability. Our results highlight the importance of accounting for survey design in causal analyses. While effect estimates were similar across methods, accounting for both confounding and selection into the observed dataset, TMLE and the G‐computation had narrower confidence intervals and may be considered suitable alternatives to the use of IPTW estimators.

## Author Contributions

L.B.‐O. and F.J.C. conceived the study, analysed and interpreted the data. Both authors participated in the drafting of the manuscript; read and approved the final version of the manuscript.

## Ethics Statement

According to Dutch law (WMO) no formal ethical review was required.

## Conflicts of Interest

The authors declare no conflicts of interest.

## Supporting information


Data S1.

Data S2.

## Data Availability

This study is based on open data from the National Health and Nutrition Examination Survey (NHANES) for the 2017–2020 cycles, which is publicly available through the Centers for Disease Control and Prevention (CDC) website [https://wwwn.cdc.gov/nchs/nhanes/continuousnhanes/default.aspx?Cycle=2017‐2020]. R code is available in the supporting information.
