# Supplementary material for: Causal Inference and Survey Data in Paediatric Epidemiology: Generalising Treatment Effects From Observational Data
Source: Paediatr Perinat Epidemiol. 2025 Jul 14;40(2):222–30. doi: 10.1111/ppe.70042 (PMC13010229; doi:10.1111/ppe.70042)
Supplement: Supplementary file 1 — Data S1. [file PPE-40-222-s002.pdf]

For the latest version of the contents of this file and future updates or resources, please visit the GitHub repository: https://github.com/LizBurgosOchoa/SurveyCausalInference

R Code to implement the approaches used in the manuscript "Causal Inference and Survey Data in Paediatric Epidemiology: Generalizing Treatment Effects from Observational Data"

L. Burgos-Ochoa and F.J. Clouth

```
# This script provides the R code to replicate the causal inference methods
# applied in the paper, including IPTW, G-computation, and TMLE with survey data.

# Load necessary libraries (make sure to have these packages installed)
library(haven)  # for reading .xpt files, the default in NHANES
library(dplyr)  # for data manipulation
library(survey) # for survey design specification and use in reg. models
library(tmle)   # TMLE package

# Data sources
# Demographic data: https://wwwn.cdc.gov/Nchs/Nhanes/2017-2018/P_DEMO.XPT
# Blood pressure data: https://wwwn.cdc.gov/Nchs/Nhanes/2017-2018/P_BPXO.XPT
# Smoking in household questionnaire data: https://wwwn.cdc.gov/Nchs/Nhanes/2017-2018/P_SMQFAM.XPT

##------------------------------------------
##------------------------------------------
#    Loading and cleaning datasets
##------------------------------------------
##------------------------------------------


#------------------------------------------
# Load NHANES Base Dataset (demographics)
#------------------------------------------
```

```
# Load NHANES Demographic data
nhanes_dem <- read_xpt(
  file = "P_DEMO.xpt",
  col_select = NULL,
  skip = 0,
  n_max = Inf,
  .name_repair = "unique"
)

cat("Number of cases:", nrow(nhanes_dem), "\n")

#-----------------------------------------
# Keep cases with MEC information
#-----------------------------------------
nhanesmec <- nhanes_dem %>%
  filter(!WTMECPRP==0)

cat("Number of cases w/ MEC data:", nrow(nhanesmec), "\n")

# Check number of PSU and Strata
unique(nhanesmec$SDMVPSU) # 3 PSU values
unique(nhanesmec$SDMVSTRA) # 24 Strata variance values

#-----------------------------------------
# Filter Data by Age Group
#-----------------------------------------

# Select cases for children aged 8-11 years
nhanesmec <- nhanesmec %>%
  filter(RIDAGEYR >= 8 & RIDAGEYR < 12)

# Verify age distribution in the filtered dataset
table(nhanesmec$RIDAGEYR)
```

```
# Check number of PSU and Strata is the same as in original dataset.
unique(nhanesmec$SDMVPSU) # 3 PSU values
unique(nhanesmec$SDMVSTRA) # 24 strata variance values

#------------------------------------------
# Load other datsets
#------------------------------------------

# Load NHANES BP data MEC Examination data
nhanes_bp <- read_xpt(
 file = "P_BPXO.xpt",
 col_select = NULL,
 skip = 0,
 n_max = Inf,
 .name_repair = "unique"
)

# Load NHANES Household Smoking Situation Questionnaire data
nhanes_smk <- read_xpt(
 file = "P_SMQFAM.xpt",
 col_select = NULL,
 skip = 0,
 n_max = Inf,
 .name_repair = "unique"
)


#------------------------------------------
# Merge Datasets and Select Relevant Variables
#------------------------------------------

# Merge demographic, physical examination, and smoking data by 'SEQN'
nhanesmec <- nhanesmec %>%
 merge(nhanes_bp, by = "SEQN", all.x = TRUE) %>%
 merge(nhanes_smk, by = "SEQN", all.x = TRUE) %>%
 # Keep relevant variables for analysis
```

```
 select(
  SEQN, RIAGENDR, RIDAGEYR, RIDRETH1, WTINTPRP, WTMECPRP, SDMVPSU, SDMVSTRA,
  INDFMPIR, BPXOSY1, BPXODI1, BPXOSY2, BPXODI2, BPXOSY3, BPXODI3, SMD460
 )

# Display the first few rows of the dataset to confirm the merge
head(nhanesmec)



cat("Number of cases merged data:", nrow(nhanesmec), "\n")

#------------------------------------------
# Create Average Blood Pressure Variables
#------------------------------------------

# Calculate average systolic blood pressure (BPXOSY1, BPXOSY2, BPXOSY3)
nhanesdat_clean <- nhanesmec %>%
 mutate(
  avg_systolic_bp = round(
   rowMeans(select(., BPXOSY1, BPXOSY2, BPXOSY3), na.rm = TRUE)
  )
 )

# Calculate average diastolic blood pressure (BPXODI1, BPXODI2, BPXODI3)
nhanesdat_clean <- nhanesdat_clean %>%
 mutate(
  avg_diastolic_bp = round(
   rowMeans(select(., BPXODI1, BPXODI2, BPXODI3), na.rm = TRUE)
  )
 )



#------------------------------------------
# Recode Variables
#------------------------------------------
```

```
# 1. Recode poverty status into categories based on INDFMPIR
#    Categories:
#    1 = Below poverty threshold (< 1.3)
#    2 = Near poverty (1.3 - 3.5)
#    3 = Above poverty threshold (> 3.5)
#    4 = Unknown

nhanesdat_clean <- nhanesdat_clean %>%
  mutate(
    poverty_cat = case_when(
      INDFMPIR < 1.3 ~ 1,
      INDFMPIR >= 1.3 & INDFMPIR <= 3.5 ~ 2,
      INDFMPIR > 3.5 ~ 3,
      TRUE ~ 4
    )
  )

# 2. Recode household smoking status based on SMD460 values
#    Categories:
#    1 = 1 or more smokers in household (SMD460 == 1 or 2), 0 = If Otherwise
nhanesdat_clean <- nhanesdat_clean %>%
  mutate(
    smk_cat = case_when(
      SMD460 == 1 ~ 1,
      SMD460 == 2 ~ 1,
      SMD460 == 0 ~ 0
    )
  )

#------------------------------------------
# Remove Cases with Missing Average BP Data
#------------------------------------------

# Count the number of cases with missing data for average BP variables
missing_cases <- nhanesdat_clean %>%
  filter(is.na(avg_systolic_bp) | is.na(avg_diastolic_bp)) %>%
```

```
  count()

# Remove cases with missing data in avg_systolic_bp or avg_diastolic_bp
nhanesdat_clean <- nhanesdat_clean %>%
  filter(!is.na(avg_systolic_bp) & !is.na(avg_diastolic_bp))

# Display the number of removed cases
cat("Number of cases removed:", missing_cases$n, "\n")



#------------------------------------------
# Remove Cases with Missing Smoking Status (smk_cat)
#------------------------------------------

# Count the number of cases with missing data in smk_cat
missing_smkcat_cases <- nhanesdat_clean %>%
  filter(is.na(smk_cat)) %>%
  count()

# Remove cases with missing data in smk_cat
nhanesdat_clean <- nhanesdat_clean %>%
  filter(!is.na(smk_cat))

# Display the number of removed cases
cat("Number of cases removed due to missing smoking status (smk_cat):", missing_smkcat_cases$n,
"\n")



# Check values for PSU and Strata, they should be the same as in original dataset.
unique(nhanesdat_clean$SDMVPSU) # 3 PSU
unique(nhanesdat_clean$SDMVSTRA) # 24 Stratification units

#------------------------------------------
# Remove variables not needed for analysis
#------------------------------------------
```

```
nhanesdat_clean <- nhanesdat_clean %>%
  select( -c(INDFMPIR, SMD460, BPXOSY1, BPXOSY2, BPXOSY3, BPXODI1, BPXODI2, BPXODI3)
  )

summary(nhanesdat_clean)

##-----------------------------------------
##-----------------------------------------
#       Analysis approaches
##-----------------------------------------
##-----------------------------------------
# Purpose: Estimate the PATE of smoking (smk_cat) on averaged diastolic and systolic blood pressure (avg_diastolic_bp & )

#---------------------------------------------
# Naive Linear Regression Model no adjustments
#---------------------------------------------

## -- Diastolic BP as outcome --##

# Define linear regression model

mod_n_dbp <- lm(
  formula = avg_diastolic_bp ~ factor(smk_cat),
  data = nhanesdat_clean
)

# model summary and 95% confidence intervals
(summary_mod_n_dbp <- summary(mod_n_dbp))
conf_intervals_n_dbp <- confint(mod_n_dbp)



## -- Systolic BP as outcome --##

# Define linear regression model
mod_n_sbp <- lm(
```

```
  formula = avg_systolic_bp ~ factor(smk_cat),
  data = nhanesdat_clean
)

# Model summary and 95% confidence intervals
(summary_mod_n_sbp <- summary(mod_n_sbp))
conf_intervals_n_sbp <- confint(mod_n_sbp)



#----------------------------------------------------------------------
# Linear Regression Model confounding adjustment, no survey weights
#----------------------------------------------------------------------

## -- Diastolic BP as outcome --##

# Define weighted linear regression model
mod_adj_dbp <- lm(
  formula = avg_diastolic_bp ~ factor(smk_cat) + factor(RIAGENDR) + factor(RIDAGEYR) +
    factor(RIDRETH1) + factor(poverty_cat),
  data = nhanesdat_clean
)

# Model summary and 95% confidence intervals
(summary_mod_adj_dbp <- summary(mod_adj_dbp))
conf_intervals <- confint(mod_adj_dbp)



## -- Systolic BP as outcome --##

# Define weighted linear regression model
mod_adj_sbp <- lm(
  formula = avg_systolic_bp ~ factor(smk_cat) + factor(RIAGENDR) + factor(RIDAGEYR) +
    factor(RIDRETH1) + factor(poverty_cat),
  data = nhanesdat_clean
)
```

```
# Model summary and 95% confidence intervals
(summary_mod_adj_sbp <- summary(mod_adj_sbp))
conf_intervals_adj_sbp <- confint(mod_adj_sbp)


#---------------------------------------------
# Linear Regression Model w/survey weights
#---------------------------------------------

# Here we use "svydesign" from the Survey package to assign the corresponding survey weights. We need the MEC weights WTMECPRP for the analysis. Additionally, "svydesign" requires information on the PSU and stratification We will use this new design variable "nhanesDesign" when running our analyses.

nhanesDesign <- svydesign(id      = ~SDMVPSU,
                strata  = ~SDMVSTRA,
                weights = ~WTMECPRP,
                nest    = TRUE,
                data    = nhanesdat_clean)


## -- Diastolic BP as outcome --##

# Define weighted linear regression model using "svyglm"
mod_svy_dbp <- svyglm(
  formula = avg_diastolic_bp ~ factor(smk_cat),
  design =  nhanesDesign
)

# Model summary and 95% confidence intervals
(summary_mod_svy_dbp <- summary(mod_svy_dbp))
conf_intervals <- confint(mod_svy_dbp)


## -- Systolic BP as outcome --##

# Define weighted linear regression model
```

```
mod_svy_sbp <- svyglm(
  formula = avg_systolic_bp ~ factor(smk_cat),
  design =  nhanesDesign
)

# Model summary and 95% confidence intervals
(summary_mod_svy_sbp <- summary(mod_svy_sbp))
conf_intervals_svy_sbp <- confint(mod_svy_sbp)




#---------------------------------------------------------------------
# Linear Regression Model w/ Survey Weights and confounding adjustment
#---------------------------------------------------------------------

# ** Note that the survey object 'nhanesDesign' has been created above.

## -- Diastolic BP as outcome --##

# Define survey weighted linear regression model adjusted by confounders

mod_svyadj_dbp <- svyglm(
  formula = avg_diastolic_bp ~ factor(smk_cat) + factor(RIAGENDR) + factor(RIDAGEYR) +
    factor(RIDRETH1) + factor(poverty_cat),
    design =  nhanesDesign
)

# Model summary and 95% confidence intervals
(summary_mod_svyadj_dbp <- summary(mod_svyadj_dbp))
conf_intervals <- confint(mod_svyadj_dbp)



## -- Systolic BP as outcome --##

# Define weighted linear regression model
```

```
mod_svyadj_sbp <- svyglm(
  formula = avg_systolic_bp ~ factor(smk_cat) + factor(RIAGENDR) + factor(RIDAGEYR) +
    factor(RIDRETH1) + factor(poverty_cat),
  design =  nhanesDesign
)

# Model summary and 95% confidence intervals
(summary_mod_svyadj_sbp <- summary(mod_svyadj_sbp))
conf_intervals_svyadj_sbp <- confint(mod_svyadj_sbp)



#-------------------------------------------------
# Inverse Probability of Treatment Weighting (IPTW)
# no Survey Weights
#-------------------------------------------------

## Step 1.Estimate Propensity Scores and calculate IPTW: this step is the same for the analyses for both of our outcomes.

# Logit Model to Estimate Propensity Scores: Define the logistic regression model to predict the probability of being exposed to smokers in the household (smk_cat).
mod_pred_A <- glm(formula = factor(smk_cat) ~ factor(RIAGENDR) + factor(RIDAGEYR) + factor(RIDRETH1) + factor(poverty_cat),
                  family = quasibinomial(), data = nhanesdat_clean)

# Add propensity scores (fitted values) to dataset and calculate IPTW
new_data_ipw <- broom::augment_columns(mod_pred_A, nhanesdat_clean, type.predict = "response") %>%
  rename(propensity = .fitted) %>%
  mutate(
    iptw = (smk_cat / propensity) + ((1 - smk_cat) / (1 - propensity))
  )


## Step 2. Run weighted regression models adjusting for IPTW values.

## -- Diastolic BP as outcome --##
```

```
# Define and fit the weighted model (iptw only) for diastolic BP

mod_iptw_dbp <- glm(formula = avg_diastolic_bp ~ factor(smk_cat), family = gaussian(), data = new_data_ipw, weights = iptw)


# Model summary and 95% confidence intervals

(summary_model_dbp_iptw <- summary(mod_iptw_dbp))

confint_model_dbp_iptw <- confint(mod_iptw_dbp)



## -- Systolic BP as outcome --##


# Define and fit the weighted model (IPTW only) for systolic BP

mod_iptw_sbp <- glm(formula = avg_systolic_bp ~ factor(smk_cat), family = gaussian(), data = new_data_ipw, weights = iptw)


# Model summary and 95% confidence intervals

(summary_model_sbp_iptw <- summary(mod_iptw_sbp))

confint_model_sbp_iptw <- confint(mod_iptw_sbp)




#--------------------------------------------------

# Inverse Probability of Treatment Weighting (IPTW)

# w/ Survey Weights

#--------------------------------------------------

# ** Note that the survey object 'nhanesDesign' has been created above.


## Step 1.Estimate Propensity Scores and calculate IPTW: this step is the same for the analyses for both of our outcomes.


# Logit Model to Estimate Propensity Scores: Define the logistic regression model to predict the probability of being exposed to smokers in the household (smk_cat).

mod_svy_pred_A <- svyglm(

  formula = factor(smk_cat) ~ factor(RIAGENDR) + factor(RIDAGEYR) + factor(RIDRETH1) + factor(poverty_cat),

  family = quasibinomial(),
```

```
  design =  nhanesDesign
)

# Add propensity scores (fitted values) to dataset and calculate IPTW
new_data_svy_ipw <- broom::augment_columns(mod_svy_pred_A, nhanesdat_clean, type.predict =
"response") %>%
  rename(propensity = .fitted) %>%
  mutate(
    iptw = (smk_cat / propensity) + ((1 - smk_cat) / (1 - propensity))
  )


## Step 2. Run weighted regression models adjusting for IPTW values.

# Add the iptw weights to the nhanesDesign object
nhanesDesign_iptw <- svydesign(id      = ~SDMVPSU,
                    strata  = ~SDMVSTRA,
                    weights = ~WTMECPRP * iptw,
                    nest    = TRUE,
                    data    = new_data_svy_ipw)

## -- Diastolic BP as outcome --##

# Define and fit the weighted model (iptw + survey weights) for diastolic BP
mod_svy_iptw_dbp <- svyglm(
  formula = avg_diastolic_bp ~ factor(smk_cat),
  design =  nhanesDesign_iptw
)

# Model summary and 95% confidence intervals
(summary_model_svy_dbp_iptw <- summary(mod_svy_iptw_dbp))
confint_model_svy_dbp_iptw <- confint(mod_svy_iptw_dbp)


## -- Systolic BP as outcome --##
```

```
# Define and fit the weighted model (IPTW + survey weights) for systolic BP
mod_svy_iptw_sbp <- svyglm(
  formula = avg_systolic_bp ~ factor(smk_cat),
  design =  nhanesDesign_iptw
)

# Model summary and 95% confidence intervals
(summary_model_svy_sbp_iptw <- summary(mod_svy_iptw_sbp))
confint_model_svy_sbp_iptw <- confint(mod_svy_iptw_sbp)



#----------------------------------------------
# G-formula (G-computation) Estimation
# no Survey Weights
#----------------------------------------------

# Set parameters
msize <- 250  # Number of Monte Carlo (MC) iterations
set.seed(234)

## -- Diastolic BP as outcome --##

# Create vectors to store results for each scenario across MC iterations
dbpmean_NC <- dbpmean_x0 <- dbpmean_x1 <- numeric(msize)

# Step 1: Fit Baseline Outcome Model

# Fit the regression model for the outcome (avg_diastolic_bp)
mod_dbp_gform <- glm(formula = avg_diastolic_bp ~ factor(smk_cat) + factor(RIAGENDR) +
factor(RIDAGEYR) +
            factor(RIDRETH1) + factor(poverty_cat), family = gaussian(), data = nhanesdat_clean)

# Step 2: Monte Carlo Simulation Loop for G-Formula

for (m in 1:msize) {
```

```
  # NATURAL COURSE (NC) Scenario
  # Predict outcomes under observed level of household smoking and covariate distribution
  sample.NC <- nhanesdat_clean
  sample.NC$dbp_sim <- rnorm(nrow(sample.NC), predict(object = mod_dbp_gform, newdata =
sample.NC), 1)
  dbpmean_NC[m] <- mean(sample.NC$dbp_sim)

  # All No Smoking (SHS = 0) Scenario
  sample_x0 <- nhanesdat_clean
  sample_x0$smk_cat <- 0  # Set smk_cat to 0 for all individuals
  sample_x0$dbp_sim <- rnorm(nrow(sample_x0), predict(object = mod_dbp_gform, newdata =
sample_x0), 1)
  dbpmean_x0[m] <- mean(sample_x0$dbp_sim)

  # All Smoking (SHS = 1) Scenario
  sample_x1 <- nhanesdat_clean
  sample_x1$smk_cat <- 1  # Set smk_cat to 1 for all individuals
  sample_x1$dbp_sim <- rnorm(nrow(sample_x1), predict(object = mod_dbp_gform, newdata =
sample_x1), 1)
  dbpmean_x1[m] <- mean(sample_x1$dbp_sim)
}


# Step 3: Calculate Average Treatment Effect (ATE)

# Calculate the mean outcome for each scenario across iterations
(mean_NC <- mean(dbpmean_NC))

# Calculate the PATE as the difference between smoking and non-smoking scenarios
(pate_gform_dbp <- mean(dbpmean_x1) - mean(dbpmean_x0))


## Step 4: Bootstrapping to obtain PATE 95% CI
set.seed(789)

## -- Diastolic BP as outcome --##
# Set parameters
```

```
bsize <- 1000  # Number of bootstrap iterations
msize <- 250  # Number of Monte Carlo iterations

# Initialize vectors to store results for each scenario across iterations
dbpmean_NC <- dbpmean_x0 <- dbpmean_x1 <- numeric(msize)
boot.pate_gform_dbp <- numeric(bsize)  # Initialize vector to store PATE for each bootstrap iteration

# Bootstrapping loop
for (bs in 1:bsize) {

 # Sample data with replacement
 boot.dat <- nhanesdat_clean[sample(1:nrow(nhanesdat_clean), size = nrow(nhanesdat_clean), replace
= TRUE), ]

 # Fit the regression model for the outcome (avg_diastolic_bp) with error handling
 mod_dbp_gform <- try({
  glm(formula = avg_diastolic_bp ~ factor(smk_cat) + factor(RIAGENDR) + factor(RIDAGEYR) +
      factor(RIDRETH1) + factor(poverty_cat), family = gaussian(), data = boot.dat)
 }, silent = TRUE)  # Suppress error messages in the console

 # Check if the model fit was successful; if not, assign NA and skip this iteration
 if (inherits(mod_dbp_gform, "try-error")) {
  boot.pate_gform_dbp[bs] <- NA  # Assign NA if there is an error
  next  # Skip to the next bootstrap iteration
 }

 # Step 2: Monte Carlo Simulation Loop for G-Formula
 for (m in 1:msize) {

  # NATURAL COURSE (NC) Scenario
  # Predict outcomes under observed level of household smoking and covariate distribution
  sample.NC <- boot.dat
  sample.NC$dbp_sim <- rnorm(nrow(sample.NC), predict(object = mod_dbp_gform, newdata =
sample.NC), 1)
  dbpmean_NC[m] <- mean(sample.NC$dbp_sim)
```

```
  # All No SHS (SHS = 0) Scenario
  sample_x0 <- boot.dat
  sample_x0$smk_cat <- 0  # Set smk_cat to 0 for all individuals
  sample_x0$dbp_sim <- rnorm(nrow(sample_x0), predict(object = mod_dbp_gform, newdata =
sample_x0), 1)
  dbpmean_x0[m] <- mean(sample_x0$dbp_sim)

  # All SHS (SHS = 1) Scenario
  sample_x1 <- boot.dat
  sample_x1$smk_cat <- 1  # Set smk_cat to 1 for all individuals
  sample_x1$dbp_sim <- rnorm(nrow(sample_x1), predict(object = mod_dbp_gform, newdata =
sample_x1), 1)
  dbpmean_x1[m] <- mean(sample_x1$dbp_sim)

 }  # End of Monte Carlo loop

 # Calculate the PATE as the difference between smoking and non-smoking scenarios
 boot.pate_gform_dbp[bs] <- mean(dbpmean_x1) - mean(dbpmean_x0)

}  # End of bootstrap loop

# Display the bootstrapped PATE results
boot.pate_gform_dbp

(ate_estimate <- mean(boot.pate_gform_dbp, na.rm=T) ) # Bootstrap mean estimate of ATE
(ci_lower <- quantile(boot.pate_gform_dbp, 0.025, na.rm=T))  # 2.5th percentile
(ci_upper <- quantile(boot.pate_gform_dbp, 0.975, na.rm=T))  # 97.5th percentile



## -- Systolic BP as outcome --##

# Set parameters
msize <- 250  # Number of Monte Carlo (MC) iterations
set.seed(234)

## -- Systolic BP as outcome --##
```

```
# Create vectors to store results for each scenario across MC iterations

sbpmean_NC <- sbpmean_x0 <- sbpmean_x1 <- numeric(msize)


# Step 1: Fit Baseline Outcome Model


# Fit the regression model for the outcome (avg_systolic_bp)

mod_sbp_gform <- glm(formula = avg_systolic_bp ~ factor(smk_cat) + factor(RIAGENDR) +
factor(RIDAGEYR) +

                factor(RIDRETH1) + factor(poverty_cat), family = gaussian(), data = nhanesdat_clean)


# Step 2: Monte Carlo Simulation Loop for G-Formula


for (m in 1:msize) {


 # NATURAL COURSE (NC) Scenario

 # Predict outcomes under observed level of household smoking and covariate distribution

 sample.NC <- nhanesdat_clean

 sample.NC$sbp_sim <- rnorm(nrow(sample.NC), predict(object = mod_sbp_gform, newdata =
sample.NC), 1)

 sbpmean_NC[m] <- mean(sample.NC$sbp_sim)


 # All No Smoking (SHS = 0) Scenario

 sample_x0 <- nhanesdat_clean

 sample_x0$smk_cat <- 0  # Set smk_cat to 0 for all individuals

 sample_x0$sbp_sim <- rnorm(nrow(sample_x0), predict(object = mod_sbp_gform, newdata =
sample_x0), 1)

 sbpmean_x0[m] <- mean(sample_x0$sbp_sim)


 # All Smoking (SHS = 1) Scenario

 sample_x1 <- nhanesdat_clean

 sample_x1$smk_cat <- 1  # Set smk_cat to 1 for all individuals

 sample_x1$sbp_sim <- rnorm(nrow(sample_x1), predict(object = mod_sbp_gform, newdata =
sample_x1), 1)

 sbpmean_x1[m] <- mean(sample_x1$sbp_sim)

}
```

```
# Step 3: Calculate Average Treatment Effect (ATE)

# Calculate the mean outcome for each scenario across iterations
(mean_NC_sbp <- mean(sbpmean_NC))

# Calculate the PATE as the difference between smoking and non-smoking scenarios
(pate_gform_sbp <- mean(sbpmean_x1) - mean(sbpmean_x0))


## Step 4: Bootstrapping to obtain PATE 95% CI
set.seed(789)

# Set parameters
bsize <- 1000  # Number of bootstrap iterations
msize <- 250  # Number of Monte Carlo iterations

# Initialize vectors to store results for each scenario across iterations
sbpmean_NC <- sbpmean_x0 <- sbpmean_x1 <- numeric(msize)
boot.pate_gform_sbp <- numeric(bsize)  # Initialize vector to store PATE for each bootstrap iteration

# Bootstrapping loop
for (bs in 1:bsize) {

  # Sample data with replacement
  boot.dat <- nhanesdat_clean[sample(1:nrow(nhanesdat_clean), size = nrow(nhanesdat_clean), replace
= TRUE), ]

  # Fit the regression model for the outcome (avg_systolic_bp) with error handling
  mod_sbp_gform <- try({
    mod_sbp_gform <- glm(formula = avg_systolic_bp ~ factor(smk_cat) + factor(RIAGENDR) +
factor(RIDAGEYR) +
                         factor(RIDRETH1) + factor(poverty_cat), family = gaussian(), data = boot.dat)
  }, silent = TRUE)  # Suppress error messages in the console

  # Check if the model fit was successful; if not, assign NA and skip this iteration
  if (inherits(mod_sbp_gform, "try-error")) {
```

```
    boot.pate_gform_sbp[bs] <- NA  # Assign NA if there is an error
    next  # Skip to the next bootstrap iteration
  }

  # Step 2: Monte Carlo Simulation Loop for G-Formula
  for (m in 1:msize) {

    # NATURAL COURSE (NC) Scenario
    # Predict outcomes under observed level of household smoking and covariate distribution
    sample.NC <- boot.dat
    sample.NC$sbp_sim <- rnorm(nrow(sample.NC), predict(object = mod_sbp_gform, newdata =
sample.NC), 1)
    sbpmean_NC[m] <- mean(sample.NC$sbp_sim)

    # All No SHS (SHS = 0) Scenario
    sample_x0 <- boot.dat
    sample_x0$smk_cat <- 0  # Set smk_cat to 0 for all individuals
    sample_x0$sbp_sim <- rnorm(nrow(sample_x0), predict(object = mod_sbp_gform, newdata =
sample_x0), 1)
    sbpmean_x0[m] <- mean(sample_x0$sbp_sim)

    # All SHS (SHS = 1) Scenario
    sample_x1 <- boot.dat
    sample_x1$smk_cat <- 1  # Set smk_cat to 1 for all individuals
    sample_x1$sbp_sim <- rnorm(nrow(sample_x1), predict(object = mod_sbp_gform, newdata =
sample_x1), 1)
    sbpmean_x1[m] <- mean(sample_x1$sbp_sim)



    print(c(bs, m))
  }  # End of Monte Carlo loop

  # Calculate the PATE as the difference between smoking and non-smoking scenarios
  boot.pate_gform_sbp[bs] <- mean(sbpmean_x1) - mean(sbpmean_x0)

}  # End of bootstrap loop
```

```
# Display the bootstrapped PATE results for Systolic BP
boot.pate_gform_sbp

# Calculate and display the mean estimate and confidence intervals
(ate_estimate_sbp <- mean(boot.pate_gform_sbp, na.rm = T))  # Bootstrap mean estimate of ATE
(ci_lower_sbp <- quantile(boot.pate_gform_sbp, 0.025, na.rm = TRUE))  # 2.5th percentile
(ci_upper_sbp <- quantile(boot.pate_gform_sbp, 0.975, na.rm = TRUE))  # 97.5th percentile



#-----------------------------------------------
# G-formula (G-computation) Estimation
# w/ Survey Weights
#-----------------------------------------------
# ** Note that the survey object 'nhanesDesign' has been created above.

# Set parameters
msize <- 250  # Number of Monte Carlo (MC) iterations
set.seed(234)

## -- Diastolic BP as outcome --##

# Create vectors to store results for each scenario across MC iterations
dbpmean_NC <- dbpmean_x0 <- dbpmean_x1 <- numeric(msize)

# Step 1: Fit Baseline Outcome Model

# Fit the regression model for the outcome (avg_diastolic_bp) using survey weights
mod_dbp_gform <- svyglm(
  formula = avg_diastolic_bp ~ factor(smk_cat) + factor(RIAGENDR) + factor(RIDAGEYR) +
    factor(RIDRETH1) + factor(poverty_cat),
  design = nhanesDesign
)

# Step 2: Monte Carlo Simulation Loop for G-Formula
```

```r
for (m in 1:msize) {

  # NATURAL COURSE (NC) Scenario
  # Predict outcomes under observed level of household smoking and covariate distribution
  sample.NC <- nhanesdat_clean
  sample.NC$dbp_sim <- rnorm(nrow(sample.NC), predict(object = mod_dbp_gform, newdata = sample.NC), 1)
  dbpmean_NC[m] <- mean(sample.NC$dbp_sim)

  # All No Smoking (SHS = 0) Scenario
  sample_x0 <- nhanesdat_clean
  sample_x0$smk_cat <- 0  # Set smk_cat to 0 for all individuals
  sample_x0$dbp_sim <- rnorm(nrow(sample_x0), predict(object = mod_dbp_gform, newdata = sample_x0), 1)
  dbpmean_x0[m] <- mean(sample_x0$dbp_sim)

  # All Smoking (SHS = 1) Scenario
  sample_x1 <- nhanesdat_clean
  sample_x1$smk_cat <- 1  # Set smk_cat to 1 for all individuals
  sample_x1$dbp_sim <- rnorm(nrow(sample_x1), predict(object = mod_dbp_gform, newdata = sample_x1), 1)
  dbpmean_x1[m] <- mean(sample_x1$dbp_sim)
}


# Step 3: Calculate Average Treatment Effect (ATE)

# Calculate the mean outcome for each scenario across iterations
(mean_NC <- mean(dbpmean_NC))

# Calculate the PATE as the difference between smoking and non-smoking scenarios
(pate_gform_dbp <- mean(dbpmean_x1) - mean(dbpmean_x0))


## Step 4: Bootstrapping to obtain PATE 95% CI
set.seed(789)
```

```
## -- Diastolic BP as outcome --##
# Set parameters
bsize <- 1000  # Number of bootstrap iterations
msize <- 250  # Number of Monte Carlo iterations

# Initialize vectors to store results for each scenario across iterations
dbpmean_NC <- dbpmean_x0 <- dbpmean_x1 <- numeric(msize)
boot.pate_gform_dbp <- numeric(bsize)  # Initialize vector to store PATE for each bootstrap iteration

# Bootstrapping loop
for (bs in 1:bsize) {

 # Sample data with replacement
 boot.dat <- nhanesdat_clean[sample(1:nrow(nhanesdat_clean), size = nrow(nhanesdat_clean), replace
= TRUE), ]

 # Define survey design with the bootstrap sample
 nhanesDesign_gboot <- svydesign(id      = ~SDMVPSU,
                       strata  = ~SDMVSTRA,
                       weights = ~WTMECPRP,
                       nest    = TRUE,
                       data    = boot.dat)

 # Fit the regression model for the outcome (avg_diastolic_bp) using survey weights with error handling
 mod_dbp_gform <- try({
  svyglm(
   formula = avg_diastolic_bp ~ factor(smk_cat) + factor(RIAGENDR) + factor(RIDAGEYR) +
    factor(RIDRETH1) + factor(poverty_cat),
   design = nhanesDesign_gboot
  )
 }, silent = TRUE)  # Suppress error messages in the console

 # Check if the model fit was successful; if not, assign NA and skip this iteration
 if (inherits(mod_dbp_gform, "try-error")) {
  boot.pate_gform_dbp[bs] <- NA  # Assign NA if there is an error
  next  # Skip to the next bootstrap iteration
```

```
  }

  # Step 2: Monte Carlo Simulation Loop for G-Formula
  for (m in 1:msize) {

    # NATURAL COURSE (NC) Scenario
    # Predict outcomes under observed level of household smoking and covariate distribution
    sample.NC <- boot.dat
    sample.NC$dbp_sim <- rnorm(nrow(sample.NC), predict(object = mod_dbp_gform, newdata =
sample.NC), 1)
    dbpmean_NC[m] <- mean(sample.NC$dbp_sim)

    # All No SHS (SHS = 0) Scenario
    sample_x0 <- boot.dat
    sample_x0$smk_cat <- 0  # Set smk_cat to 0 for all individuals
    sample_x0$dbp_sim <- rnorm(nrow(sample_x0), predict(object = mod_dbp_gform, newdata =
sample_x0), 1)
    dbpmean_x0[m] <- mean(sample_x0$dbp_sim)

    # All SHS (SHS = 1) Scenario
    sample_x1 <- boot.dat
    sample_x1$smk_cat <- 1  # Set smk_cat to 1 for all individuals
    sample_x1$dbp_sim <- rnorm(nrow(sample_x1), predict(object = mod_dbp_gform, newdata =
sample_x1), 1)
    dbpmean_x1[m] <- mean(sample_x1$dbp_sim)

  }  # End of Monte Carlo loop

  # Calculate the PATE as the difference between smoking and non-smoking scenarios
  boot.pate_gform_dbp[bs] <- mean(dbpmean_x1) - mean(dbpmean_x0)

}  # End of bootstrap loop

# Display the bootstrapped PATE results
boot.pate_gform_dbp

(ate_estimate <- mean(boot.pate_gform_dbp, na.rm=T) ) # Bootstrap mean estimate of ATE
```

```
(ci_lower <- quantile(boot.pate_gform_dbp, 0.025, na.rm=T))  # 2.5th percentile
(ci_upper <- quantile(boot.pate_gform_dbp, 0.975, na.rm=T))  # 97.5th percentile


## -- Systolic BP as outcome --##

# Set parameters
msize <- 250  # Number of Monte Carlo (MC) iterations
set.seed(234)

## -- Systolic BP as outcome --##

# Create vectors to store results for each scenario across MC iterations
sbpmean_NC <- sbpmean_x0 <- sbpmean_x1 <- numeric(msize)

# Step 1: Fit Baseline Outcome Model

# Fit the regression model for the outcome (avg_systolic_bp) using survey weights
mod_sbp_gform <- svyglm(
  formula = avg_systolic_bp ~ factor(smk_cat) + factor(RIAGENDR) + factor(RIDAGEYR) +
    factor(RIDRETH1) + factor(poverty_cat),
  design = nhanesDesign
)

# Step 2: Monte Carlo Simulation Loop for G-Formula

for (m in 1:msize) {

  # NATURAL COURSE (NC) Scenario
  # Predict outcomes under observed level of household smoking and covariate distribution
  sample.NC <- nhanesdat_clean
  sample.NC$sbp_sim <- rnorm(nrow(sample.NC), predict(object = mod_sbp_gform, newdata =
sample.NC), 1)
  sbpmean_NC[m] <- mean(sample.NC$sbp_sim)

  # All No Smoking (SHS = 0) Scenario
```

```
  sample_x0 <- nhanesdat_clean
  sample_x0$smk_cat <- 0  # Set smk_cat to 0 for all individuals
  sample_x0$sbp_sim <- rnorm(nrow(sample_x0), predict(object = mod_sbp_gform, newdata =
sample_x0), 1)
  sbpmean_x0[m] <- mean(sample_x0$sbp_sim)

  # All Smoking (SHS = 1) Scenario
  sample_x1 <- nhanesdat_clean
  sample_x1$smk_cat <- 1  # Set smk_cat to 1 for all individuals
  sample_x1$sbp_sim <- rnorm(nrow(sample_x1), predict(object = mod_sbp_gform, newdata =
sample_x1), 1)
  sbpmean_x1[m] <- mean(sample_x1$sbp_sim)
}

# Step 3: Calculate Average Treatment Effect (ATE)

# Calculate the mean outcome for each scenario across iterations
(mean_NC_sbp <- mean(sbpmean_NC))

# Calculate the PATE as the difference between smoking and non-smoking scenarios
(pate_gform_sbp <- mean(sbpmean_x1) - mean(sbpmean_x0))


## Step 4: Bootstrapping to obtain PATE 95% CI
set.seed(789)

# Set parameters
bsize <- 1000  # Number of bootstrap iterations
msize <- 250  # Number of Monte Carlo iterations

# Initialize vectors to store results for each scenario across iterations
sbpmean_NC <- sbpmean_x0 <- sbpmean_x1 <- numeric(msize)
boot.pate_gform_sbp <- numeric(bsize)  # Initialize vector to store PATE for each bootstrap iteration

# Bootstrapping loop
for (bs in 1:bsize) {
```

```
 # Sample data with replacement
 boot.dat <- nhanesdat_clean[sample(1:nrow(nhanesdat_clean), size = nrow(nhanesdat_clean), replace
= TRUE), ]

 # Define survey design with the bootstrap sample
 nhanesDesign_gboot <- svydesign(id      = ~SDMVPSU,
                        strata  = ~SDMVSTRA,
                        weights = ~WTMECPRP,
                        nest    = TRUE,
                        data    = boot.dat)

 # Fit the regression model for the outcome (avg_systolic_bp) using survey weights with error handling
 mod_sbp_gform <- try({
  svyglm(
   formula = avg_systolic_bp ~ factor(smk_cat) + factor(RIAGENDR) + factor(RIDAGEYR) +
    factor(RIDRETH1) + factor(poverty_cat),
   design = nhanesDesign_gboot
  )
 }, silent = TRUE)  # Suppress error messages in the console

 # Check if the model fit was successful; if not, assign NA and skip this iteration
 if (inherits(mod_sbp_gform, "try-error")) {
  boot.pate_gform_sbp[bs] <- NA  # Assign NA if there is an error
  next  # Skip to the next bootstrap iteration
 }

 # Step 2: Monte Carlo Simulation Loop for G-Formula
 for (m in 1:msize) {

  # NATURAL COURSE (NC) Scenario
  # Predict outcomes under observed level of household smoking and covariate distribution
  sample.NC <- boot.dat
  sample.NC$sbp_sim <- rnorm(nrow(sample.NC), predict(object = mod_sbp_gform, newdata =
sample.NC), 1)
  sbpmean_NC[m] <- mean(sample.NC$sbp_sim)
```

```
   # All No SHS (SHS = 0) Scenario
   sample_x0 <- boot.dat
   sample_x0$smk_cat <- 0  # Set smk_cat to 0 for all individuals
   sample_x0$sbp_sim <- rnorm(nrow(sample_x0), predict(object = mod_sbp_gform, newdata =
sample_x0), 1)
   sbpmean_x0[m] <- mean(sample_x0$sbp_sim)

   # All SHS (SHS = 1) Scenario
   sample_x1 <- boot.dat
   sample_x1$smk_cat <- 1  # Set smk_cat to 1 for all individuals
   sample_x1$sbp_sim <- rnorm(nrow(sample_x1), predict(object = mod_sbp_gform, newdata =
sample_x1), 1)
   sbpmean_x1[m] <- mean(sample_x1$sbp_sim)

  }  # End of Monte Carlo loop

  # Calculate the PATE as the difference between smoking and non-smoking scenarios
  boot.pate_gform_sbp[bs] <- mean(sbpmean_x1) - mean(sbpmean_x0)

}  # End of bootstrap loop

# Display the bootstrapped PATE results for Systolic BP
boot.pate_gform_sbp

# Calculate and display the mean estimate and confidence intervals
(ate_estimate_sbp <- mean(boot.pate_gform_sbp, na.rm = T))  # Bootstrap mean estimate of ATE
(ci_lower_sbp <- quantile(boot.pate_gform_sbp, 0.025, na.rm = TRUE))  # 2.5th percentile
(ci_upper_sbp <- quantile(boot.pate_gform_sbp, 0.975, na.rm = TRUE))  # 97.5th percentile




#-----------------------------------------------
# Targeted Maximum Likelihood Estimation (TMLE)
# no Survey Weights
#-----------------------------------------------
```

Step 1.Estimate Propensity Scores, this step is the same for the analysis for both outcomes.

```
# Define formula for regression model to predict exposure level, i.e., smk_cat
txmodel <- "smk_cat ~ factor(RIAGENDR) + factor(RIDAGEYR) + factor(RIDRETH1) + factor(poverty_cat)"

# Make a copy of the dataset to work with
sampled.data <- nhanesdat_clean

# Calculate propensity scores for each individual in the sample
sampled.data$pscore <- predict(
  glm(formula = txmodel, family = quasibinomial(), data = sampled.data),
  type = "response"
)
```

Step 2: Define Covariate Matrix (W) for TMLE, this step is shared by the analyses for both of our outcomes

```
W <- as.data.frame(cbind(
  W1 = factor(sampled.data$RIAGENDR),
  W2 = factor(sampled.data$RIDAGEYR),
  W3 = factor(sampled.data$RIDRETH1),
  W4 = factor(sampled.data$poverty_cat)
))
```

Step 3: Run TMLE Model and obtain PATE point estimate

```
set.seed(789)

## -- Diastolic BP as outcome --##

# Fit TMLE to estimate the effect of smk_cat on avg_diastolic_bp
# Using 10000 bootstrap iterations to obtain the 95% CI, B=10000

mod_tmle_dbp <- tmle(
  Y = sampled.data$avg_diastolic_bp,
```

```
  A = sampled.data$smk_cat,
  W = W,
  Qform = "Y ~ A + W1 + W2 + W3 + W4",
  g1W = sampled.data$pscore,
  B=10000
)

# PATE estimate
mod_tmle_dbp$estimates$ATE$psi

# 95% CI, percentile method
mod_tmle_dbp$estimates$ATE$bs.CI.twosided



## -- Systolic BP as outcome --##

# Fit TMLE to estimate the effect of smk_cat on avg_systolic_bp
# Using 10000 bootstrap iterations to obtain the 95% CI, B=10000
mod_tmle_sbp <- tmle(
  Y = sampled.data$avg_systolic_bp,
  A = sampled.data$smk_cat,
  W = W,
  Qform = "Y ~ A + W1 + W2 + W3 + W4",
  g1W = sampled.data$pscore,
  B=10000
)

# PATE estimate for Systolic BP
mod_tmle_sbp$estimates$ATE$psi

# 95% CI for Systolic BP, percentile method
mod_tmle_sbp$estimates$ATE$bs.CI.twosided



#-----------------------------------------------
```

```
# Targeted Maximum Likelihood Estimation (TMLE)
# w/ Survey Weights
#-----------------------------------------------
## Step 1.Estimate Propensity Scores, this step is the same for the analysis for both outcomes.

# Define formula for regression model to predict exposure level, i.e., smk_cat
txmodel <- "smk_cat ~ factor(RIAGENDR) + factor(RIDAGEYR) + factor(RIDRETH1) + factor(poverty_cat)"

# Make a copy of the dataset to work with
sampled.data.svy <- nhanesdat_clean
nhanesDesign_tmle <- svydesign(id      = ~SDMVPSU,
                    strata  = ~SDMVSTRA,
                    weights = ~WTMECPRP,
                    nest    = TRUE,
                    data    = sampled.data.svy)

# Calculate propensity scores for each individual in the sample
sampled.data.svy$pscore.svy <- predict(
  svyglm(formula = txmodel, family = quasibinomial(), design = nhanesDesign_tmle),
  type = "response"
)


## Step 2: Define Covariate Matrix (W) for TMLE, this step is shared by the analyses for both of our outcomes
W_svy <- as.data.frame(cbind(
  W1 = factor(sampled.data.svy$RIAGENDR),
  W2 = factor(sampled.data.svy$RIDAGEYR),
  W3 = factor(sampled.data.svy$RIDRETH1),
  W4 = factor(sampled.data.svy$poverty_cat)
))


## Step 3: Run TMLE Model and obtain PATE point estimate
set.seed(789)
```

```
## -- Diastolic BP as outcome --##

# Fit TMLE to estimate the effect of smk_cat on avg_diastolic_bp
# Using 10000 bootstrap iterations to obtain the 95% CI, B=10000

mod_tmle_svy_dbp <- tmle(
  Y = sampled.data.svy$avg_diastolic_bp,
  A = sampled.data.svy$smk_cat,
  W = W_svy,
  Qform = "Y ~ A + W1 + W2 + W3 + W4",
  g1W = sampled.data.svy$pscore.svy,
  obsWeights = sampled.data.svy$WTMECPRP, B=10000
)

# PATE estimate
mod_tmle_svy_dbp$estimates$ATE$psi

# 95% CI, percentile method
mod_tmle_svy_dbp$estimates$ATE$bs.CI.twosided



## -- Systolic BP as outcome --##

# Fit TMLE to estimate the effect of smk_cat on avg_systolic_bp
# Using 10000 bootstrap iterations to obtain the 95% CI, B=10000
mod_tmle_svy_sbp <- tmle(
  Y = sampled.data.svy$avg_systolic_bp,
  A = sampled.data.svy$smk_cat,
  W = W_svy,
  Qform = "Y ~ A + W1 + W2 + W3 + W4",
  g1W = sampled.data.svy$pscore.svy,
  obsWeights = sampled.data.svy$WTMECPRP, B=10000
)

# PATE estimate for Systolic BP
```

```
mod_tmle_svy_sbp$estimates$ATE$psi


# 95% CI for Systolic BP, percentile method
mod_tmle_svy_sbp$estimates$ATE$bs.CI.twosided
```
