# Supplementary material for: Causal Inference and Survey Data in Paediatric Epidemiology: Generalising Treatment Effects From Observational Data
Source: Paediatr Perinat Epidemiol. 2025 Jul 14;40(2):222–30. doi: 10.1111/ppe.70042 (PMC13010229; doi:10.1111/ppe.70042)
Supplement: Supplementary file 2 — Data S2. [file PPE-40-222-s001.docx]

## Causal Inference and Survey Data in Paediatric Epidemiology: Generalizing Treatment Effects from Observational Data

Lisbeth Burgos-Ochoa Felix J. Clouth

## Supplemental Material

| 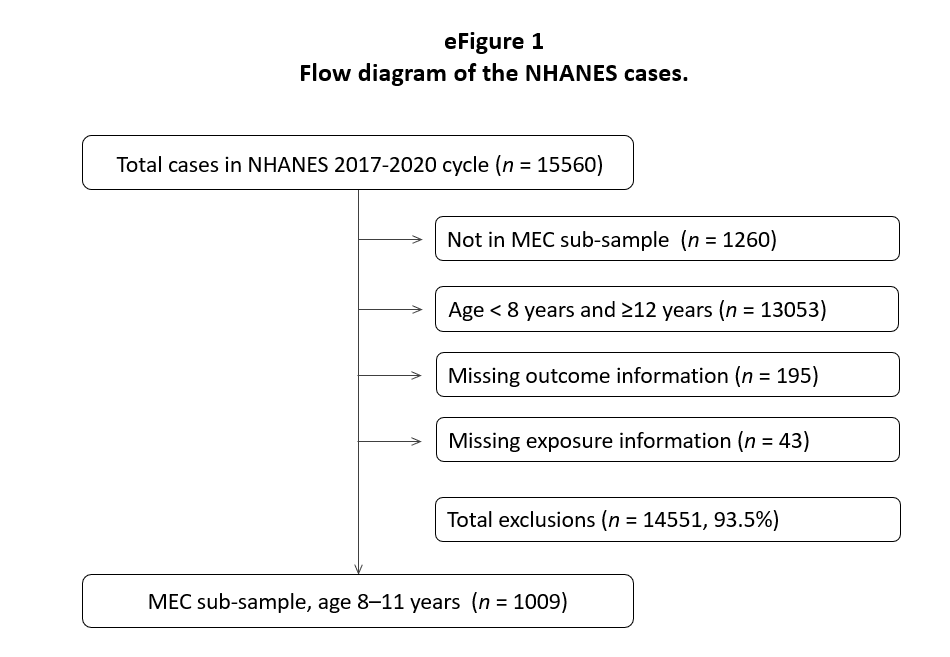 |
| --- |
| **eFigure 1.** Flow diagram of the NHANES cases.  Footnote: MEC refers to the Mobile Examination Center of the NHANES survey, where physical measurements, including blood pressure, are conducted. Only participants in the MEC sub-sample have outcome data for this analysis. |

| **eTable 1. Technical description of the use of sample weights in the three estimators on interest.** |
| --- |
| Survey selection weights (sample weights) are defined as $\omega^{\Delta_{\mathrm{svy}}=1}=\frac{1}{P(\Delta_{\mathrm{svy}}=1\vert\boldsymbol{W})}$ where $\Delta_{\mathrm{svy}}$ is a binary variable indicating inclusion in the survey sample and $\boldsymbol{W}$ is a vector of known covariates determining inclusion in the survey sample.^24^ In this section, we refer to $\boldsymbol{L}$ as the set of covariates used in the analysis to adjust for confounding.  To implement these sample weights in an IPTW analysis, a new set of weights is estimated as $\omega^{SHS=1,\Delta_{\mathrm{svy}}=1}=\frac{1}{\pi_{i}}*\omega^{\Delta_{\mathrm{svy}}=1}$ and $\omega^{SHS=0,\Delta_{\mathrm{svy}}=1}=\frac{1}{{1-\pi}_{i}}*\omega^{\Delta_{\mathrm{svy}}=1}$.^10^ Note that the propensity scores are estimated on individuals that were included in the sample, hence, $\pi_{i}$ are probabilities conditional on $\Delta_{\mathrm{svy}}=1$. The PATE can then be estimated as the marginal difference in weighted outcome between the exposure groups $E\left( Y*\omega^{SHS=1,\Delta_{\mathrm{svy}}=1}\vert SHS=1 \right)-E\left( Y*\omega^{SHS=0,\Delta_{\mathrm{svy}}=1}\vert SHS=0 \right)$.  For the g-formula, estimate of the PATE, we follow the steps presented in the previous section. We use the sample weights $\omega^{\Delta_{\mathrm{svy}}=1}$ to fit a model for $E\left( Y*\omega^{\Delta_{\mathrm{svy}}=1} \vert SHS,\boldsymbol{L} \right)$ on the sample for which we observe $Y$. Setting exposure to its corresponding values, counterfactual outcomes are obtained by means of Monte Carlo (MC) simulation. The difference between these counterfactual outcomes is then calculated and the PATE is obtained by taking the weighted sum of these differences across observations in the sample using the sample weights $\sum\left[ Y_{shs=1}-Y_{shs=0} \right]$.  The TMLE estimate of the PATE is obtained in a similar fashion. Counterfactual outcomes are obtained using the same procedure as in the g-formula. These counterfactual outcome are then updated using the covariate $H$ which now uses our new analysis weights such that $H_{i}={shs}_{i}*\omega^{shs=1,\Delta_{\mathrm{svy}}=1}-\left( 1-{shs}_{i} \right)*\omega^{shs=0,\Delta_{\mathrm{svy}}=1}$.^10^ Again, the PATE is then obtained by taking the weighted sum of these differences across observations in the sample using the sample weights. |

| **eTable 2. Variables in NHANES analysis.** |
| --- |
| Outcomes During the physical examination in MECs, systolic and diastolic BPs (SBP and DBP) were measured using three readings. We used the average of all available readings.^4^ The outcomes corresponded to continuous DBP and SBP in mmHg. Exposure For this analysis, household SHS exposure was assessed using data from the household smoking questionnaire, which documented the smoking status of household members. This variable was chosen due to the missing observations other SHS indicators, such as cotinine levels (from urine samples). Although for the purpose of this illustration household smoking status is used as exposure, in applied research, incorporating additional SHS indicators would be preferable. The exposure variable was recoded as 1 if one or more household members smoked, and 0 otherwise. Covariates Information on various covariates was obtained from the demographic dataset from NHANES. These include sex, age (in years), ethnicity, and the poverty-to-income ratio (PIR), which measures family income relative to the federal poverty level. For ethnicity, participants were categorized as Mexican American, Other Hispanic, Non-Hispanic White, Non-Hispanic Black, and Other. The PIR was recoded into four categories: below the poverty threshold (PIR < 1.3), near poverty (PIR between 1.3 and 3.5), above the poverty threshold (PIR > 3.5), and unknown (for unavailable PIR values). |

| **eTable 3. Numerical results for the estimates of the effect of SHS on BP.** | | |
| --- | --- | --- |
| **Estimator** | **Diastolic Blood Pressure (95% CI)** | **Systolic Blood Pressure (95% CI)** |
| Naive Regression | 0.26 (-0.77, 1.29) | 0.14 (-1.04, 1.31) |
| Regression (Sample Weights Only) | 0.95 (-0.55, 2.45) | 0.52 (-1.55, 2.60) |
| Regression (Confounding Adjusted Only) | 0.12 (-0.96, 1.20) | -0.54 (-1.75, 0.67) |
| Regression (Confounding & Sample Weights) | 0.53 (-1.06, 2.11) | -0.20 (-2.23, 1.83) |
| IPTW | 0.14 (-0.81, 1.10) | -0.68 (-1.77, 0.41) |
| IPTW (Sample Weights) | 0.69 (-1.01, 2.39) | -0.25 (-2.50, 2.01) |
| G-Formula | 0.12 (-0.90, 1.20) | -0.54 (-1.78, 0.72) |
| G-Formula (Sample Weights) | 0.54 (-0.73, 1.92) | -0.19 (-1.86, 1.38) |
| TMLE | 0.12 (-1.04, 1.32) | -0.74 (-2.01, 0.55) |
| TMLE (Sample Weights) | 0.63 (-0.78, 2.04) | -0.33 (-2.01, 1.34) |
